# A brave new, but frightening world

**DOI:** 10.4103/0971-3026.76042

**Published:** 2011

**Authors:** Bhavin Jankharia

**Affiliations:** Editor-in-Chief, The Indian Journal of Radiology and Imaging “F”, 1^st^ Floor, Bhaveshwar Vihar, 383, Sardar V P Road, Mumbai - 400 004, India. E-mail: editor@ijri.org

Last month’s editorial[1] kicked up quite a storm. Two readers’ responses have been published as Letters in this issue,[2,3] while a few more, will be published in the May 2011 issue.

The world has changed. It no longer follows the simpler, linear trajectory that our senior radiologists were used to. Enter medical college, pass out, join radiology, pass out, join a hospital or start a practice with an X-ray machine (ultrasound came a little later), provide good service, earn a decent living, make friends and colleagues, attend conferences and then retire or die with your boots on.

The coming of ultrasound in the 1980s changed things a bit, because now there was a slightly larger investment to be made. However, this technology was still affordable; there was no PNDT (Pre-Natal Sex Determination Act) and there was no competition from gynecologists, vascular surgeons, urologists and “whoever else thinks it’s easy to wear a transducer” physician.

Things started changing the 1990s, when individual radiologists turned entrepreneurs and started installing their own CT scanners and MRIs. What was once the domain of large hospitals, soon became a field that was taken over by enterprising private radiologists. With the investments rising and the stakes becoming higher, it became necessary to understand financial issues, debt, equity, depreciation, return on investment (ROI), internal rate of return (IRR), return on capital employed (ROCE) and similar other terms. Negotiations that were once only with equipment vendors, spilt over to bankers, venture capitalists, angel investors, private equity firms, third party lease operators and the like.

In the early 2000s, once access to capital became easy and the younger radiologists saw that it wasn’t difficult to start their own high-end imaging centers, competition grew as well. This has led to radiologists learning about innovate marketing, sales strategies, public relations (PR) campaigns, web-based eyeball grabbing, footfall evaluation, customer relationship management (CRM) initiatives, etc. Receptionists have become front-desk executives, typists have become transcriptionists and we have added on personal patient managers, floor managers and even tea and water delivery executive managers.

Statutory compliance is suddenly the new “buzz” word. Now, we need to be tax compliant, Atomic Energy Regulatory Board (AERB) compliant, PNDT compliant, shops and establishment compliant, fire compliant, insurance compliant, water purity compliant, waste disposal compliant, signboard compliant, and I guess eventually compliant enough to be able to bend down and touch our toes and leave our backsides unprotected to the world to do whatever it wants to.

Larger centers have organizational charts and structures. Suddenly, the head of the department (HOD) has become the Chief Executive Officer (CEO). The accountant is now the CFO (Chief Financial Officer). And suddenly all kinds of titles have started sprouting up with CIOs, CTOs, CPOs and even CBOs (Information, Technology, Productivity and Belief, respectively). We have managers to handle quality, administration, stationery, procurement, logistics, supply-chain, hardware, software, networking … there is no end to this madness.

This is more than we asked for when we decided to take up radiology as a discipline. The practice of radiology today involves the “business of radiology” as an integral part of the discipline. It is as important to know about tax issues and codes, as it is to know how to diagnose appendicitis on USG and CT scan with confidence. Though some of us are lucky to be able to devote more than 80% of our times to the practice of clinical radiology, a good number of radiologists, willingly or unwillingly need to spend time away from the core clinical practice of the discipline that they’ve chosen, so that they can focus and concentrate on the business, the administration and other similar necessary issues. Some revel in it, but I suspect the vast majority does it only because it does not have a choice.

Do send in your Letters-to-the-Editor on this subject, well in advance if you would like to perhaps see it published in the IJRI.

This issue apart from the interesting Letters-to-the-Editor also showcases a wide variety of articles dealing with technical issues in Interventional Radiology, along with two excellent pictorial essays, one on craniosynostosis[4] and the other on childhood urethral abnormalities.[5]
